# Next‐generation conservation genetics and biodiversity monitoring

**DOI:** 10.1111/eva.12661

**Published:** 2018-07-17

**Authors:** Margaret E. Hunter, Sean M. Hoban, Michael W. Bruford, Gernot Segelbacher, Louis Bernatchez

**Affiliations:** ^1^ U.S. Geological Survey Wetland and Aquatic Research Center Gainesville Florida; ^2^ The Morton Arboretum Lisle Illinois; ^3^ Cardiff School of Biosciences and Sustainable Places Institute Cardiff University Cardiff UK; ^4^ Wildlife Ecology and Management University Freiburg Freiburg Germany; ^5^ GIROQ Département de Biologie Université Laval Ste‐Foy, Québec QC Canada

**Keywords:** adaptive management, effective population size, genetic diversity, minimally invasive sampling, RAD‐seq, single nucleotide polymorphisms

## Abstract

This special issue of *Evolutionary Applications* consists of 10 publications investigating the use of next‐generation tools and techniques in population genetic analyses and biodiversity assessment. The special issue stems from a 2016 Next Generation Genetic Monitoring Workshop, hosted by the National Institute for Mathematical and Biological Synthesis (NIMBioS) in Tennessee, USA. The improved accessibility of next‐generation sequencing platforms has allowed molecular ecologists to rapidly produce large amounts of data. However, with the increased availability of new genomic markers and mathematical techniques, care is needed in selecting appropriate study designs, interpreting results in light of conservation concerns, and determining appropriate management actions. This special issue identifies key attributes of successful genetic data analyses in biodiversity evaluation and suggests ways to improve analyses and their application in current population and conservation genetics research.

Modern molecular techniques provide unprecedented power to understand genetic variation in natural populations. Nevertheless, application of this information requires sound understanding of population genetics theory. Fred Allendorf (2017)

## 1 INTRODUCTION

As biodiversity loss accelerates and environmental challenges mount, there is a need for quantitative evaluation of the status and trends of intraspecific and interspecific genetic diversity of species and communities. Assessing variation in neutral and adaptive loci can identify genetic threats to populations, species, and communities (Alsos et al., 2012; Hemingway et al., 2018). Such assessments can also help to identify the precise mechanism of diversity loss (e.g., correlated with habitat fragmentation; Jump, Hunt, & Peñuelas, 2006; Vranckx, Jacquemyn, Muys, & Honnay, 2012) and which human activities most impact the genetic variation and evolutionary potential of the species (Aguilar, Quesada, Ashworth, Herrerias‐Diego, & Lobo, 2008; DiBattista, 2008; Hoban et al., 2010). By monitoring genetic diversity through time, we can determine long‐term impacts and assess whether interventions have met conservation targets and improved biodiversity (this issue, Flanagan, Forester, Latch, Aitken, & Hoban, 2018; Hoban et al., 2014).

Recent technological advances have enabled routine assessment of genetic diversity at the genome level (Garner et al., 2016; Narum, Buerkle, Davey, Miller, & Hohenlohe, 2013). However, as genetic datasets are becoming larger and more complex, and analyses are becoming more specialized, thoughtful project planning and application of statistical tools are increasingly needed. Inappropriate choice of study design or analysis can lead to incorrect conclusions, and thus misguided interventions (Lotterhos & Whitlock, 2015; Meirmans, 2015). Moreover, there is recognition that currently available analyses do not make full use of large genomic datasets (Lotterhos et al., 2017; Villemereuil, Frichot, Bazin, François, & Gaggiotti, 2014), and that both informatic and theoretical advances are still needed. These improvements to genetic monitoring and analyses are the focus of this special issue.

At the time of writing, the proposed Convention on Biological Diversity 2020 Aichi Biodiversity Targets is 2 years away. These targets were developed in Aichi, Japan, in 2010 and provide an overarching framework for the United Nations system and various Nations and Conventions to preserve biodiversity (https://www.cbd.int/sp/targets/). These targets are focused on integrating biodiversity awareness, valuation, and measurements into governmental and societal action; they are designed to safeguard ecosystems, species, and genetic diversity against the loss of biodiversity by promoting sustainable processes and management. Specifically, Target 13 states that by 2020 “the genetic diversity of cultivated plants and farmed and domesticated animals and of wild relatives, *including other socioeconomically as well as culturally valuable species* [emphasis added], is maintained, and strategies have been developed and implemented for minimizing genetic erosion and safeguarding their genetic diversity.”

In many species, this target has not yet been met, as studies continue to document genetic erosion across many animal and plant taxa (e.g., Laikre et al., 2010; Nielsen, Gebhard, Smalla, Bones, & van Elsas, 1997; Vilà et al., 2003; Zhu et al., 2013). However, there are increasing efforts to safeguard genetic resources of wild and domesticated plants and animals in situ and ex situ (Mounce, Smith, & Brockington, 2017; O'Donnell & Sharrock, 2017). Policies such as the Global Strategy for Plant Conservation and Montreal Process on Sustainable Forest Management are also emphasizing the measurement and sustainability of genetic diversity. Still, there remains an almost complete lack of “genetic indicators” or direct measures at the genetic level to quantify progress toward this target in wild species, with the sole reported indicator being the proportion of livestock breeds at risk of extinction (Tittensor et al., 2014). In short, the preservation of genetic diversity in wild systems is well recognized in theory but less so in practice, partly due to a need for better‐applied conservation genetics tools and guidance for implementation in management decisions.

Until approximately 2010, much of the phylogenetic, evolutionary applications, and conservation genetic analyses were conducted using PCR on between one and 20 loci, or electrophoresis on >30 allozymes. Most studies involved Sanger sequencing at a handful of nuclear markers or mitochondrial DNA loci, or fragment analysis of ~10–20 microsatellites. As we transition into the next phase of genomic analysis, individuals and populations can be assessed at 1,000s to millions of loci using next‐generation sequencing (NGS). This massively parallel high‐throughput sequencing approach produces high coverage sequencing reads for many loci and samples (Andrews, Good, Miller, Luikart, & Hohenlohe, 2016). Comparisons with traditional markers have indicated that NGS datasets tend to provide finer scale resolution and additional biological insights, such as evidence of adaptive diversity or deleterious mutations (Ferchaud, Laporte, Perrier, & Bernatchez, 2018; Fuentes‐Pardo & Ruzzante, 2017; Perrier, Ferchaud, Sirois, Thibault, & Bernatchez, 2017). Subsequently, third‐generation sequencing, often referred to as long‐read sequencing, now allows for direct sequencing of large DNA fragments, which has advantages in de novo genome assemblies and metagenomics (Fuentes‐Pardo & Ruzzante, 2017). In addition to the sheer quantity of data becoming available via NGS platforms, novel data types can be assessed, such as investigating functional loci, regulatory motifs, and metabarcoding datasets.

Due to improved accessibility of NGS platforms, molecular ecologists can efficiently produce large amounts of data compared to traditional markers, but careful consideration is needed to decide whether the cost and resources are warranted. Guidelines could help improve the transition between traditional legacy datasets collected over many years and new NGS datasets, including when to transition to a new DNA marker system. In some instances, new information could affect conservation decisions and/or endangered species status listings that have already been made (e.g., Oyler‐McCance, Cornman, Jones, & Fike, 2015). Further, with the availability of numerous types of genomic markers and mathematical tools for testing specific hypotheses, care is needed in selecting appropriate study designs (Forester, Lasky, Wagner, & Urban, 2018). Power analyses and simulations (i.e., Hoban, 2014) are being developed to determine the appropriate number of samples and density of markers for genomewide genotyping analyses such as pedigree reconstruction, minimally invasive sampling (MIS), genome scans to identify loci under selection, and species delineations (e.g., Catchen et al., 2017). Additionally, data interpretation must be based on concrete population‐level mechanisms, as improper interpretations of model assumptions or data from new sequencing techniques could lead to incorrect inferences (Schuster, 2008). Therefore, increasing levels of expertise and awareness regarding the strengths and shortcomings of new methods are required for bioinformatic analyses.

In November 2016, a Next Generation Genetic Monitoring Workshop was hosted by the National Institute for Mathematical and Biological Synthesis (NIMBioS) in Tennessee, USA. The goal of the workshop was to help unlock the conservation potential of genomic data for biodiversity studies and lay a foundation for describing, quantifying, and interpreting the complex, multidimensional information contained in new theories and approaches. Next‐generation sequence data analyses will need to be integrated among various research areas such as noninvasive sampling, taxonomic delineations, landscape genetics, forensics, microbiomics, and epigenetic studies as well as interdisciplinary fields such as climate science, phenotypic analysis, and geospatial remote sensing. The workshop therefore included experts comprising empiricists, theoreticians, and method developers from divergent geographic areas, genders, career stages, and expertise to spark cross‐disciplinary discussions. This special issue synthesizes the contributions and discussions made at the workshop to assist those working with NGS data for conservation and management.

The workshop participants identified key attributes of successful data analysis in biodiversity evaluation and surveyed and critiqued existing genetic metrics to improve analyses and how they might be applied to current needs. For example, monitoring tools should be able to assess system conditions, diagnose the cause of population or diversity losses (e.g., harvest or habitat fragmentation), and predict future changes. Moreover, they should ideally be easily measured, simple to apply, readily understood by nonspecialists such as decision makers, and respond to stressors in a predictable manner (Dale & Beyeler, 2001). Due to the complexity of genomic studies, many of the customary statistical methods do not fit these criteria.

Discussion among participants exposed several areas that warrant further development of tools, experimentation, model development, and theoretical integration. Approaches were also identified for summarizing and translating highly dimensional genetic data for interpretation by natural resource managers and policymakers. The workshop was held in November of 2016 during a time when scientific and conservation funding was decreasing in some countries, while increasing in others. Discussions were conducted with the recognition that this is a sensitive time for conservation biology and our global society overall. The field of population genetics will need to embrace these challenges and transition to focus on global conservation priorities.

This special issue stems from collaborations made by a diverse array of population geneticists, mathematicians, and bioinformaticians focusing on several key questions in the field today. How many loci and samples are needed for accurate statistical analyses? How will analyses need to change to correctly interpret the data? How can ecologists and evolutionary biologists find common analytical ground? How and when does one transition from traditional markers to NGS?

## 2 CONTENT OF THE SPECIAL ISSUE

As we monitor the loss of genetic diversity using genomic analyses, it is important to understand the requirements of marker density and sample size for accurate evolutionary interpretation and management determinations. Leroy et al. (2018) evaluated the quantitative metrics used to monitor genetic erosion using NGS data and found that the appropriate number of markers and samples largely depended on population demography, statistical metrics, and the tested hypothesis. Unlike previous broadly applicable “rule of thumb” recommendations in population genetics (i.e., 30 individuals per population), investigators using NGS must carefully choose a study design, which can take advantage of prior information on population demography and information from traditional genetic markers.

Gaughran et al. (2018) conducted an empirical study comparing microsatellite markers with single nucleotide polymorphisms (SNPs) in the Galapagos giant tortoise species complex (*Chelonoidis* spp.), with the goal of detecting genetic differentiation among three species. The authors found similar results using both marker types, as long as genetic groups were correctly partitioned based on evolutionary signals. Using >20,000 SNPs, only two to five individuals were required to obtain accurate population differentiation measures with F_st_ estimates, although incorrect grouping of samples led to a lack of population structure. Thus, while theoretically a small number of individuals can be sufficient, it may be wise to use a larger number to reduce incorrect interpretation of groupings. Further examining the relationship between traditional markers and genomic data, Ferchaud et al. (2018) conducted an empirical study on populations of stocked Lake Trout (*Salvelinus namaycush*) by comparing microsatellites to SNP data. The two marker types produced similar results, but the ~5,000 SNPs also allowed for investigations into adaptation and deleterious mutations, improving the evidence available for management decisions. In particular, the joint identification of neutral and deleterious mutations could help refine the choice of source and sink supplementations to maximize evolutionary potential and limit mutation load.

Carroll et al. (2018) target the interface between MIS methods and NGS approaches. Minimally invasive samples are often opportunistically collected from the environment and consequently contain limited amounts of DNA, which can restrict the subsequent molecular analyses of these samples, especially when applying NGS methods. The authors provide guidance on how to transition legacy datasets of microsatellites or mitochondrial DNA to genomic platforms and integrate novel methods such as microbiome and epigenetic studies. Swift et al. (2018) utilized MIS methods and multifaceted DNA metabarcoding (MDM) to obtain six different data classes of genetic information from bat guano. Next‐generation sequencing was used to collect data on bat species composition, individual genotypes, sex ratios, diet, parasites, and the presence of White‐nose syndrome. The study provided information on the six data classes that were consistent with single data class analyses. To ensure high detection rates across the assays, the authors advise testing the accuracy of a broad range of primers with varying taxonomic resolution.

Regarding genetic analysis, Jost et al. (2018) provide a description and comparison of two complementary measures of population structure which are sometimes confused in the literature: fixation indices and allelic differentiation metrics. Fixation measures (G_st_, F_st_, and theta) estimate proximity to fixation in demes as opposed to the degree of differentiation of allele frequencies. The authors use several simple examples to address the misconception that the two metrics are correcting or estimating one another and demonstrated that this assumption can yield invalid inferences. For example, allelic differentiation measures may be more useful in some conservation situations when the relative sizes of the demes differ. And Jost's D, a heterozygosity‐based measurement, was found to be informative to assess genetic divergence between populations. The authors also discuss the importance of considering locus mutation rate, number of alleles, and whether loci are under selection, when using both sets of statistics.

As molecular methods change and expand, model assumptions must be carefully investigated to ensure that inferences are reflective of population processes. Milligan et al. (2018) focused on expanding methods for independent inference of local dispersal and population density. The spatial Λ‐Fleming‐Viot (SLFV) model is well suited to decouple population parameter estimates from the processes that define population structure. This is important because it is well known that different genetic processes can lead to the same signal in the data (i.e., fragmentation and population expansion). A variety of population structures can be examined to determine the most plausible. To investigate the interaction between contemporary effective population size (N_e_) and demographic population size (N_c_), Pierson, Graves, Banks, Kendall, and Lindenmayer (2018) used long‐term demographic and genetic datasets to determine whether trends in N_e_ and N_c_ accurately reflect one another. Of the four case studies examined, only two indicated a strong correlation, suggesting that more work is needed to determine when approaches to estimate N_e_ are reliable and appropriate. Parameters such as mating system and thresholds for rare allele frequencies influenced estimates and could result in incorrect interpretations without careful consideration. Overall, while N_e_ analyses can accurately reflect reduced N_e_/N_c_, the estimated values may be biased in certain situations; a point that is often lost when translating results into management applications.

Building on studies addressing local adaptation, Flanagan et al. (2018) provided an adaptive management framework for natural resource managers to determine when and how genomic tools should be employed to detect and preserve local adaptation. The authors conclude that genomic datasets may be informative in some cases, while selectively neutral genetic markers or even common garden experiments may be more efficient in others. Guidelines are provided for study design, interpretation, and application in management decisions. The authors argue for a need for strong supporting evidence from field and laboratory studies, and well‐annotated genomes for locus identification. Overall, the authors emphasize that genomic studies may reveal genetic diversity of adaptive value, but in many cases, it will be too soon to make management decisions based solely on signatures of adaptation. Gaggiotti et al. (2018) present a unifying framework for the assessment of biodiversity measurements using Hill numbers, a family of measures that provide estimates of the effective number of species present in an assemblage, and differ only in the relative importance they assign to rare species. These diversity measures are used to describe complex spatial hierarchical structures bridging molecular, population, species, and ecosystem levels. The use of the framework is demonstrated using a coral reef biodiversity dataset. By synthesizing the information at all ecosystem levels, biodiversity studies can be better integrated across different fields like conservation biology, community ecology, and incorporating eco‐evolutionary dynamics for management.

The papers in this issue highlight exciting new opportunities for using next‐generation data to provide affordable and comprehensive tools for studying populations. The use of NGS will allow the number of genetic markers to be scaled up by orders of magnitude and will promote a much greater understanding of the genetic composition of populations and individuals. Additionally, such studies increase our understanding of functional genetic processes through the investigation of adaptive loci and the expression pattern of specific genes. This special issue articulates the promise of new tools for population genetics and demonstrates the potential of these new tools. It also emphasizes, however, that limitations and uncertainties persist regarding the most appropriate analyses, techniques, interpretations, and implementations.

Looking forward, challenges in the field of population genetics include improvements in archiving data, both in the capabilities to house large volumes of data and in the public deposition of published data (many journals now require data archiving). Critically, translation and communication of genetic results and implications for natural resource managers still need improvement. For example, N_e_ values are most accurate in idealized population simulations, but may be misleading in the management of real‐world populations when used as a direct proxy for population size estimates. Similarly, the inference of the adaptive potential of given populations, while becoming feasible through NGS assessment of adaptive loci, still needs careful and informed interpretation when applied to evaluate ecosystem functions (see Ferchaud et al., 2018 and Flanagan et al., 2018). Another major advancement of our time, the development of genetic manipulation technologies in combination with gene drive systems, presents the opportunity to modify organisms in a radically new way. To date, this has been primarily applied in the control of diseases, such as producing infertile mosquitos to prevent malaria transmission (Eckhoff, Wenger, Godfray, & Burt, 2017; Gantz et al., 2015; Hammond et al., 2016). In the future, additional options will include introducing genetic variation in imperiled species to recover lost genotypes, improve diversity, reduce inbreeding, or improve resistance to specific diseases (Piaggio et al., 2017). Continued development of guidelines and experimental investigations into the feasibility and utility of these new synthetic biology‐based approaches are needed (Akbari et al., 2015; Oye et al., 2014).

Following the NIMBioS workshop, there was a meeting of the IUCN (International Union for the Conservation of Nature) Conservation Genetics Specialist Group (CGSG) in November 2017 hosted by the Antwerp Zoo, Belgium. Members from 14 countries outlined priorities for both wild and cultivated species in the fields of conservation genetics and applied evolutionary studies. The incorporation of genetic diversity and conservation unit delineation into the assessment of species for assignment to the IUCN Red List of Threatened Species was identified as an urgent need. Detailed protocols are also needed for appropriate monitoring of changes in genetic diversity; specifically, there is still no genetic indicator for Target 13 except for domesticated livestock. Simple and easily interpreted indicators are urgently needed to complete the upcoming the 2020 Targets. The group also recognized that the potential of genomic information is still largely overlooked in policy and conservation management. To address these issues, the CGSG has identified several guidelines to help managers understand the applicability of population genetic studies and better decide when genomic tools would improve conservation outcomes. Lastly, the CGSG has committed to development of guidelines for genetic considerations in Key Biodiversity Areas (sites contributing significantly to the global persistence of biodiversity; IUCN, 2016), further engaging with global policy makers such as the IUCN World Congress, and additional training and collaboration. The papers presented in this special issue form the scientific basis for many of these guidelines. It is vital that genetic information is included in the political decision‐making processes aimed at halting and reversing biodiversity loss at national and global scales.

We would like to dedicate this special issue to Dr. Tim King of the US Geological Survey, Leetown Science Center. Dr. King was a renowned conservation geneticist, making key contributions to the field and promoting the application of genetic data in management decisions. His focus, detail, and thoughtful approach to science paved the way for many of us by highlighting the importance of genetic data in imperiled and invasive species management. Dr. King was also a beloved friend and mentor to many and we endeavor to carry his extraordinary legacy forward.
